# Effectiveness of Using Mobile Technology to Improve Cognitive and Social Skills Among Individuals With Autism Spectrum Disorder: Systematic Literature Review

**DOI:** 10.2196/20892

**Published:** 2021-09-28

**Authors:** Phil Wai Shun Leung, Shirley Xin Li, Carmen Sze Oi Tsang, Bellavista Long Ching Chow, William Chi Wai Wong

**Affiliations:** 1 Department of Family Medicine and Primary Care Li Ka Shing Faculty of Medicine The University of Hong Kong Hong Kong China (Hong Kong); 2 Rehabilitation Services Haven of Hope Christian Service Hong Kong China (Hong Kong); 3 Department of Psychology Faculty of Social Sciences The University of Hong Kong Hong Kong China (Hong Kong); 4 The State Key Laboratory of Brain and Cognitive Sciences The University of Hong Kong Hong Kong China (Hong Kong)

**Keywords:** autism spectrum disorder, mobile devices, systematic review, randomized controlled trial, social skills, cognitive skills

## Abstract

**Background:**

Mobile technology has become a necessity in the lives of people in many countries. Its characteristics and advantages also make it a potential medium of intervention for people with autism spectrum disorder (ASD).

**Objective:**

The objective of this review was to evaluate previous evidence, obtained in randomized controlled trials (RCTs), on the effectiveness of using mobile devices as the medium of intervention targeting social and cognitive skills among individuals with ASD.

**Methods:**

Literature search was conducted on electronic databases including Medline, PsycInfo, PsycArticles, Education Resources Information Centre, and Social Science Citation Index. Only RCTs published in English and after year 2000 were included for this review. Data extraction was carried out by 2 independent reviewers using constant comparative methods.

**Results:**

Totally 10 RCTs were identified. Most of the findings indicated that mobile devices could be an effective medium of intervention for people with ASD, among which 6 indicated significant intervention effects and 2 showed mixed findings. Effective intervention was more likely to be achieved in the studies that recruited older participants (aged over 9 years), targeting practical skills that could be readily applied in real life, or using pictures or materials that were highly relevant in daily life in the apps or mobile devices. Furthermore, the use of mobile devices was also reported to promote participation in the intervention among individuals with ASD.

**Conclusions:**

The results suggested that mobile devices could be a promising means for the delivery of interventions targeting people with ASD. Although including a small number of studies was a limitation of this review, the results provided useful implications for designing effective mobile technology–assisted interventions for the ASD population in future studies.

## Introduction

Individuals with autism spectrum disorder (ASD) face difficulties in the social and cognitive domains of their lives. According to the fifth edition of the Diagnostic and Statistical Manual of Mental Disorders [1], the 2 core symptoms of ASD include limitations in social interaction and communication, together with stereotypic interests and behaviors. These limitations create significant problems when functioning in social situations including friendship building and daily interaction with others, in vocational or school settings that require effective problem-solving skills and persistence, and in daily life situations where flexibility and the ability to accept changes are necessary. Furthermore, difficulties in mood regulation create extra difficulties for carers and professionals to provide effective training and treatments, which could further perpetuate the aforementioned problems.

In recent years, the rapidly increasing popularity of mobile technology has provided a new possibility for providing interventions. Mobile technology refers to any handheld digital devices including different brands of mobile phones or smartphones, such as iPod touch, personal digital assistants (PDAs), or tablet computers. The World Health Organization statistics suggest that the number of mobile phone subscriptions reached 6.9 billion in 2014 [2], which is believed to continue increasing. High accessibility, together with the versatility owing to the wide variety of apps available on the market [3], makes mobile technology an effective medium of intervention. Mobile devices also offer additional advantages such as greater flexibility, lower costs, and overcoming geographical limitations when compared with traditional face-to-face treatment. Several characteristics of mobile technology such as the highly attractive screen and stimulating visual display, high portability [4], entertaining music and game functions [5], ease of use even for people with disabilities [4,6], and potential usage in augmentative and alternative communication, regarded as “a general term for communication support encompassing low-tech and high-tech systems,” [5] appear to be particularly suitable for use among people with ASD.

There are 3 main approaches in using mobile technology as the medium of intervention for individuals with ASD in previous research. First, tailored apps serve as the main tools of intervention. For example, King and her colleagues developed the Proloquo2Go app to train individuals in requesting skills [3]. Another app called iTake Turns was designed to train for turn-taking behaviors [7], whereas the MyTalk mobile software installed in iPod Touch was used to train for functional communication [5]. All these studies showed positive training results. Second, mobile devices are used as speech-generating devices (SGDs) [8-10]. In short, the SGDs facilitate communication by transferring what the users have selected (ie, touching objects on the screen) to an audible output from the devices. Previous research has successfully employed SGDs to improve various communication skills among people with ASD, such as requesting continuation of toy play [8], 3-step communication sequences (ie, general request for toys, specific request for a selected toy, followed by a thank you response) [9], and training for mand repertoire [10]. Finally, some interventions use video modeling or video prompting strategies to deliver training through mobile devices, which have been shown to successfully train for transitional behaviors in schools [11], vocational and daily living skills [12], multiple-step job performance [13], and handwashing skills [14].

According to the weak central coherence hypothesis [15], people with ASD tend to focus on details but fail to capture the meaning of a global picture during information processing. They are unable to see the link between situations or environments, so this tendency makes it difficult for them to generalize their newly learned skills across different settings. Despite these difficulties, previous systematic reviews have shown that mobile technology–based interventions targeting people with ASD could produce significant improvements in the acquisition of mand or functional communication repertoire [16] and academic skills [17]. Hong and colleagues reported similar positive results in their systematic review of single case studies on the effectiveness of tablet-mediated interventions for people with ASD [18]. Recently, Moon and his colleagues conducted a systematic review with meta-analysis to evaluate the effectiveness of mobile device intervention in randomized controlled trials (RCTs) [19]. The meta-analysis showed positive results, suggesting that mobile interventions could significantly improve performance in the fine motor and visual areas. Collectively, the findings showed that mobile technology could be an effective and attractive means of intervention for people with ASD.

Although previous systematic reviews on various interventional case studies reported promising results, one limitation was that most of these reviewed studies employed single case designs or a multiple probe design or were based on a small sample (fewer than 10 subjects). RCTs in this area have increased in recent years. Even though the review conducted by Moon and his colleagues [19] provided positive evidence concerning the effectiveness of mobile intervention in people with ASD, a more detailed investigation where the characteristics of these trials are particularly useful would be crucial, as the findings could provide useful practical implications for designing future RCTs in this area. As a result, this systematic review focuses on currently available RCT studies with the following specific research question: What are the crucial characteristics that contribute to effective mobile intervention to promote social and cognitive skills among people with ASD?

## Methods

### Sources of Data

Literature search was conducted on electronic databases of multiple disciplines, including Medline, PsycInfo, PsycArticles, Education Resources Information Centre, and Social Science Citation Index. This selection covered the widely used databases in the education, psychology, medicine, and social science fields. Additional internet searches were carried out to identify relevant articles that could answer the above research question. The search terms used during these searches were “Autis* or Asperger* or pervasive development* disorder* (Title)” and “mobile* or apps* or tablet* or iPad* or iPod* or handheld*(Title);” the 2 first authors conducted initial screening of the titles and abstracts of the identified studies.

### Eligibility and Exclusion Criteria

Inclusion criteria for this systematic review were interventional studies targeting people with ASD using touch-screen mobile devices published in English. To include studies with higher levels of evidence (Level 1b as described by the Centre for Evidence-Based Medicine [20]), this review only focused on studies with RCT designs and excluded those with case studies or other noninterventional studies such as cross-sectional surveys, cohorts, reviews, or discussion papers. Given that the popularity of mobile phones and tablets has only been increasing in recent years, only articles published from January 1, 2000, to March 31, 2019, were selected [17,21].

### Procedures

The preparation of this systematic review was in accordance with the PRISMA (Preferred Reporting Items for Systematic Review and Meta-Analysis) guidelines [22]. The first authors (LPWS and WWCW) conducted the literature search using the keywords listed above. After removing all the duplicated articles, initial screening of the titles and abstracts was carried out to ensure the identified studies met the inclusion and exclusion criteria. Finally, 2 independent researchers (LPWS and BLLC) conducted a more thorough examination of the full texts of the eligible studies to investigate the final eligibility.

### Risk of Bias Assessment

The Cochrane risk of bias tool [23] is the most frequently used tool to evaluate the quality of RCTs [24] and has the advantage of measuring possible biases in the selected studies including selection, performance, detection, attribution, reporting, and other types of biases. RevMan 5 (Cochrane Training) [25] was used to summarize and generate statistics of the assessment results.

### Data Extraction and Analysis

From the selected studies, the following categories of data were extracted: basic information of the articles (author, title, year of publication, journal, and country); participants’ details (eg, age, gender, and intelligence level); treatment content and treatment format employed (eg, mobile phone, apps, and tablets); characteristics of intervention delivery (eg, frequency, duration, and group size); description of intervention in the control group; outcome measures; and effectiveness of the treatment (defined by any significant improvements in primary outcomes). Two independent reviewers carried out the extraction of data and reached consensus after discussion on the discrepancies. Using constant comparative methods, a third independent reviewer’s opinion would be sought for any disagreement between the 2 reviewers.

In this review, the selected studies had a variety of treatment goals and employed different assessment methods, such as using game scores in apps, observing actual performances, or calculating scores using validated scales. Therefore, meta-analysis might not a feasible way of data analysis as the small number may be prone to providing misleading conclusions. Instead, the findings of the current study were based on the analysis of the descriptive summaries of the selected studies.

Specifically, interventions would be classified as either “effective,” “partially effective,” or “ineffective” based on the outcome evaluations. Similar to the data extraction procedures, 2 reviewers conducted the classification independently, together with a third reviewer when there was any disagreement. More specifically, effective interventions meant that the main targeted outcomes were found to improve significantly after the intervention; “partially effective” denoted interventions in which some (but fewer than half) indicators of the primary outcome showed significant improvement, whereas “ineffective” interventions meant the data of the targeted outcomes could not justify the intervention as effective. Satisfactory reliability of this classification system was achieved, as the level of agreement between reviewers reached 100%.

## Results

### Article Inclusion and Exclusion

**Figure 1:**
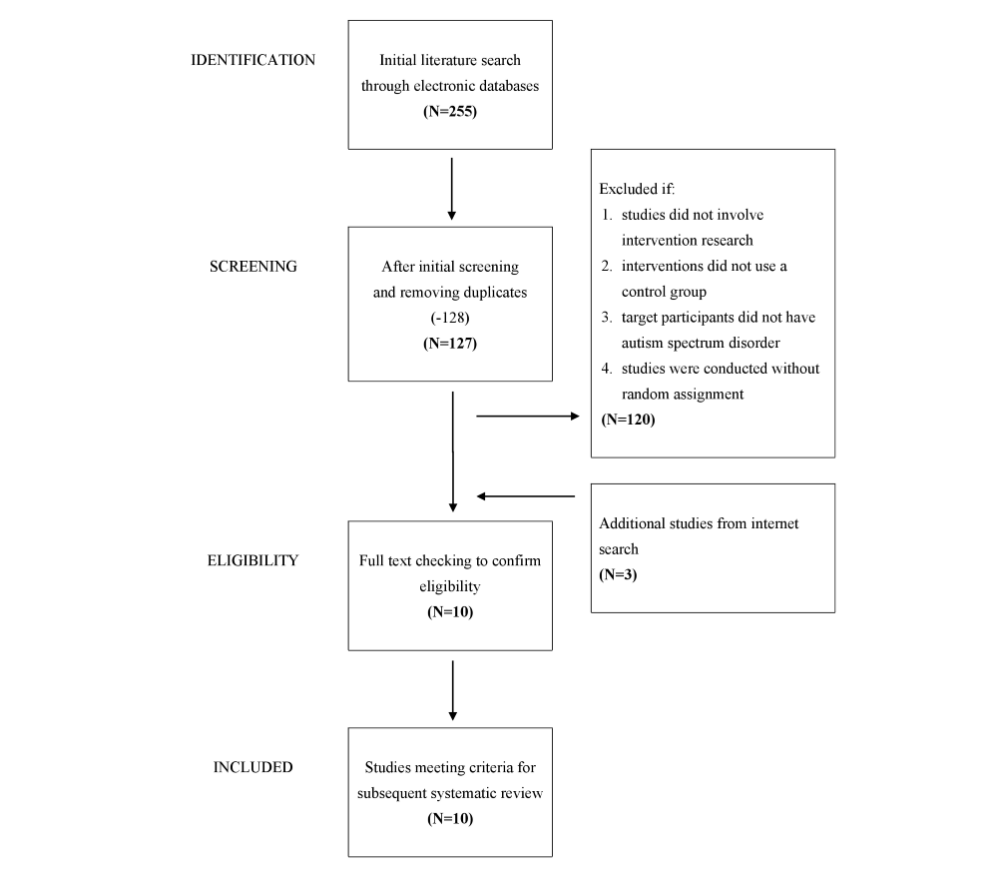
PRISMA (Preferred Reporting Items for Systematic Review and Meta-Analysis) checklist for systematic review.

The initial search identified a total of 255 articles, among which 128 articles were excluded owing to duplication, and a further 120 articles were excluded as they did not meet the inclusion criteria (ie, not interventional studies, not recruiting participants with ASD, or intervention studies that did not employ an RCT design). A further Google search identified 3 more articles that met the inclusion criteria. Finally, this systematic review included a total of 10 articles [26-35]. Figure 1 shows the flowchart of this process.

### Risk of Bias Assessment

**Figure 2:**
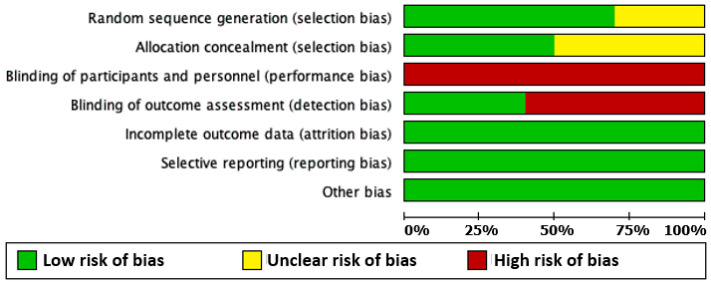
Overall risk of bias assessment results among the 10 included studies.

**Figure 3:**
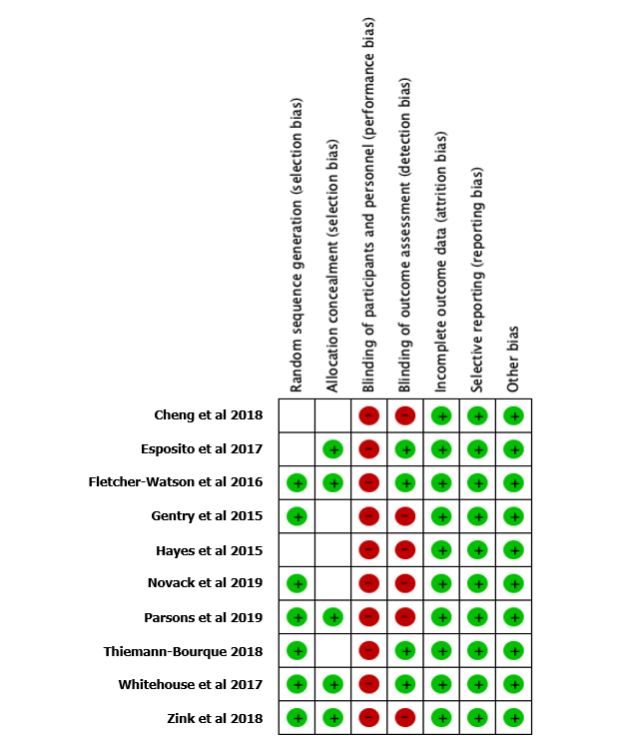
Detailed risk of bias assessment results in individual studies.

Figures 2 and 3 show the risk of bias assessment results. Figure 2 gives an overall description of the biases among the 10 included studies, whereas Figure 3 provides a more detailed explanation of the bias assessment results in individual studies.

Although all the studies adopted random assignment for group allocation, 3 studies failed to give sufficient details on how the randomization was conducted [26-28] and 5 did not describe the procedure of allocation concealment [26,28-31]. In addition, given the different interventions in experimental and control groups, it was impossible to blind the participants and personnel in these studies. Only 4 studies were able to blind the assessors to minimize assessment bias [27,31-33]. Finally, all the included studies had low risk of bias for incomplete data, selective reporting, and other biases.

### Overview of Included Studies

Table 1 provides a summary of the research designs used in the 10 selected studies.

Social skills were selected as the main treatment outcomes in half of these studies [26,27,31,33,34]. Among these, 2 studies targeted improving the cognitive skills of people with ASD using mobile technology [29,30]. In the remaining 3 studies, the intervention targeted cognitive and social skills among children with ASD [27,32,35].

**Table 1:** Characteristics of the research design in the studies included in this review (N=10).

Characteristic			n (%)
**Age of participants**			
	Below 6 years (toddlers to young children)	6 (60)	
	Between 9 and 18 years (children to adolescents)	3 (30)	
	Above 18 years	1 (10)	
**Total number of participants with ASD^a^**			
	30 participants or fewer	4 (40)	
	31 to 60 participants	5 (50)	
	61 to 100 participants	1 (10)	
**Duration** **of intervention**			
	1 month or shorter	4 (40)	
	2 to 3 months	3 (30)	
	Longer than 3 months	2 (20)	
	Not specified	1 (10)	
**Format of intervention**			
	In-session training	3 (30)	
	Used in their own time (whenever convenient or necessary)	7 (70)	
**Experimental group content**			
	Self-constructed app	8 (80)	
	PDA^b^-based app	1 (10)	
	Speech-generating device	1 (10)	
**Control group content**			
	Treatment as usual (including delayed treatment)	6 (60)	
	Picture exchange communication system	1 (10)	
	Behavioral therapy	1 (10)	
	Paper-based emotion card training	1 (10)	
	Use of untrained peers	1 (10)	
**Outcome variables**			
	Social skills	5 (50)	
	Cognitive skills	2 (20)	
	Cognitive and social skills	3 (30)	

^a^ASD: autism spectrum disorder

^b^PDA: personal digital assistant

Most of the interventions targeted young children or adolescents, 6 of which recruited children under the age of 6 years [27,30-33,35]; 3 of them targeted those aged 9 to 18 years [26,28,34], and only 1 study recruited adults with ASD [29]. Findings suggested that interventions targeting those aged 9 years or above were all found effective (all the 4 studies considered), but the effectiveness was less robust among younger children, as shown in Table 2 (2 effective, 2 partially effective, and 2 ineffective).

**Table 2:** Effectiveness across different characteristics selected in the trials (N=10).

Characteristic		Effectiveness^a^		
		Effective, n (%)	Partially effective^b^, n (%)	Ineffective, n (%)
**Age of participants**				
	Below 6 years (n=6)	2 (33)	2 (33)	2 (33)
	Between 9 to 18 years (n=3)	3 (100)	0 (0)	0 (0)
	Above 18 years (n=1)	1 (100)	0 (0)	0 (0)
**Duration of intervention**				
	1 month or shorter (n=4)	3 (75)	0 (0)	1 (25)
	2 to 3 months (n=3)	2 (67)	0 (0)	1 (33)
	Longer than 3 months (n=2)	1 (50)	1 (50)	0 (0)
**Format** **of intervention**				
	In-session training (n=3)	3 (100)	0 (0)	0 (0)
	Used in their own time (n=7)	3 (43)	2 (29)	2 (29)
**Control group** **content**				
	Treatment as usual (including delayed treatment) (n=6)	3 (50)	2 (33)	1 (17)
	Picture exchange communication system (n=1)	1 (100)	0 (0)	0 (0)
	Behavioral therapy (n=1)	0 (0)	0 (0)	1 (100)
	Paper-based emotion card training (n=1)	1 (100)	0 (0)	0 (0)
	Use of untrained peers (n=1)	1 (100)	0 (0)	0 (0)
**Outcome variables**				
	Social skills (n=5)	4 (80)	0 (0)	1 (20)
	Cognitive skills (n=2)	2 (100)	0 (0)	0 (0)
	Social and cognitive skills (n=3)	0 (0)	2 (67)	1 (33)

^a^Effectiveness is defined by the extent of significant improvements in the selected primary outcomes of the studies.

^b^Partially effective means that fewer than half of the indicators of the main outcomes showed significant improvement.

The sample sizes in these studies were generally small, with 4 studies recruiting 30 or fewer participants [26-28,30], and the sample size in the other 5 studies ranged between 31 and 60 participants [29,31,33-35]. The duration of the interventions ranged from 3 weeks to 6 months, and there were studies with long and short durations that reported insignificant intervention effects.

Given the advantage of high accessibility to mobile technology devices, over half of the studies (7/10) provided the training devices to participants who could use them at any time convenient to them [26-29,32,33,35], whereas in the other 3 studies, only in-session trainings were delivered through mobile devices at a fixed duration and frequency [30,31,34]. Nevertheless, all the studies using in-session training were found to be effective (3 out of 3 studies, Table 2).

In terms of interventional content, the authors of 8 studies developed their own apps as the training content was tailored to their training objectives [26-28,30,32-35]. In the remaining 2 studies [29,31], the authors used the built-in functions of the mobile devices to design their training, namely the speech-generating functions to facilitate communication [31], and the built-in PDA-based apps to provide vocational support [29]. As for the content of the control groups, more than half of them employed treatment as usual or delayed treatment design [28-30,32,33,35].

### Elements of Effective Interventions

Table 3 summarizes each of the 10 selected studies.

**Table 3:** Summary of the 10 selected trials.

Authors	Mean age of participants	Duration	Intervention content	Format	Control	Main outcomes (operational definitions and tools)	Effectiveness
Esposito et al [27]	47 months	4 weeks	App games: a game play app to train for vocabulary, attention, and imitation skills	Self-use	Behavioral therapy	Cognitive and social skills: vocabulary, attention, and imitation skills (1) Target improvement (2) Game scores	Ineffective: (1) Attention: *P*=.06 Imitation of actions with objects: *P*=.84. Receptive identification of objects: *P*=.21 (2) Attention: *P*=.47 Imitation of actions with objects: *P*=.02 Receptive identification of objects: *P*=.32
Thiemann- Bourque et al [31]	Exptl gp^a^: 48 months Ctl gp^b^: 46 months Peers: 40 to 61 months	9 to 19 weeks	SGD^c^ with trained peers: promoting communication through SGD and trained peers, guided by trained school staff	In-session training	Untrained peers	Social skills: communication behaviors (1) Rate of communication (2) Balanced initiation and response during interactions	Effective: (1) Rate of communication: *P*<.001 (2) Balanced initiation and response; *P*=.047
Fletcher- Watson et al [33]	Exptl gp: 49.30 months Ctl gp: 49.96 months	2 months	FindMe app: a game play app to train for attending to people (touching the single person on the screen) and following social cues (touch the item being pointed and looked at)	Self-use	TAU^d^	Social skills: social communication skills (1) BOSCC^e^ (2) ADOS-2^f^ (3) MCDI^g^ (4) CSBS-DP^h^	Ineffective: (1) Overall: *P*=.29 Social communication: *P*=.56 (2) Communication: *P*=.93 Reciprocal social interaction: *P*=.40 Social effect: *P*=.52 Restricted repetitive behavior: *P*=.81 (3) Words understood: *P*=.12 Words produced: *P*=.09 Gestures: *P*=.07 (4) Social communication: *P*=.31 Gestures: *P*=.93
Parsons et al [35]	Exptl gp: 64.4 months Ctl gp: 60.8 months	3 months	TOBY^i^ app: a game play app to train for visual motor (perception and discrimination of sensory information), imitation (copy an action), language (recognize and reproduce object and action names), and social skills (eye gaze, gestures, etc)	Self-use	Delayed treatment	Cognitive and social skills: visual motor, imitation, language, and social skills MSEL^j^ CSBSMSEL^k^ ToPMSEL^l^ POMMSEL^m^ SPTMSEL^n^	Partially effective: (1) Visual reception: *P*=.39 Fine motor: *P*=.15 Receptive language: *P*=.04 Expressive language: *P*=.30 (2) Social domain: *P*<.001 Speech domain: *P*=.07 Symbolic domain: *P*<.001 (3) ToP: *P*=.12 (4) POM: *P*=.02 (5) SPT: *P*=.95
Novack et al [30]	69.29 months	4 weeks	Camp Discovery app: identifying the correct words based on the instructions they heard	In-session training	Delayed treatment	Cognitive skills: receptive language skills (1) Number of new target words learned	Effective (1) Number of words learned: *P*<.001
Whitehouse et al [32]	3.38 years	6 months	TOBY app: a game play app to train for visual motor (perception and discrimination of sensory information), imitation (copy an action), language (recognize and reproduce object and action names), and social skills (eye gaze, gestures, etc)	Self-use	TAU	Cognitive and social skills: early behavioral intervention (1) ATEC^o^ (2) MSEL (3) VABS-II^p^	Partially effective: (1) Total: *P*=.14 Communication: *P*=.08 Sociability: *P*=.50 Sensory: *P*=.20 Physical: *P*=.28 (2) Receptive language: *P*=.32 Expressive language: *P*=.89 Visual reception: *P*=.03 Fine motor: *P*=.07 (3) Total composite: *P*=.08 Communication: *P*=.10 Socialization: *P*=.35 Daily living skills: *P*=.03 Motor skills: *P*=.08
Cheng et al [26]	Exptl gp: 11.3 years Ctl gp: 10.9 years	3 weeks	3DFER^q^ app: identifying facial expressions and the associations between situations and emotions	Self-use	Paper- based	Social skills: emotional facial recognition skills (1) Achievement scores in the app game	Effective (1) Posttest between group score difference: *P*<.001
Zink et al [34]	Exptl gp: 12.5 years Ctl gp: 12.0 years	Not described	App with self-constructed pictures: showing dental procedures with images and audio comments	In-session training	PECS^r^	Social skills: patient-dentist communication (1) Number of attempts required for skill acquisition (2) Number of dental appointments required to complete dental cleaning	Effective (1) Number of attempts required for skills acquisition: *P*<.001 (2) Number of dental appointments required: *P*<.001
Hayes et al [28]	All aged 18 years (except one aged 17)	1 month	VidCoach app: use of video modeling and video prompting strategies to learn interview skills	Self-use	TAU	Social skills: job interview skills (1) Scores in interview performance assessed by employers	Effective (1) Scores improved in intervention group: *P*<.001
Gentry et al [29]	24 years	3 months	PDA^s^ in iPod: Training on iPod Touch–based apps and strategies to support their job	Self-use	Delayed treatment	Cognitive skills: job coaching support (1) Cumulative job coaching hours (2) Monthly job coaching hours	Effective (1) Difference in cumulative coaching hours: *P*<.001 (2) Difference in monthly coaching hours: *P*<.001

^a^Exptl gp: experimental group.

^b^Ctl gp: control group.

^c^SGD: speech-generating device

^d^TAU: treatment as usual.

^e^BOSCC: Brief Observation of Social Communication Change.

^f^ADOS-2: Autism Diagnostic Observation Schedule second edition.

^g^MCDI: MacArthur Communicative Development Inventory.

^h^CSBS-DP: Communication and Symbolic Behavior Scale – Developmental Profile.

^i^TOBY: Therapy Outcomes By You.

^j^MSEL: Mullen Scales of Early Learning.

^k^CSBS: Communication and Symbolic Behavior Scale.

^l^ToP: Test of Playfulness.

^m^POM: Pragmatic Observation Measure.

^n^SPT: Symbolic Play Test.

^o^ATEC: Autism Treatment Evaluation Checklist.

^p^VABS-II: Vineland Adaptive Behavior Scale second edition.

^q^3DFER: 3D complex facial expression recognition.

^r^PECS: picture exchange communication system.

^s^PDA: personal digital assistant.

In the 2 studies that used real-life pictures or 3D animations in the apps [26,34], the intervention was effective. These materials highly resembled what the participants could encounter in daily life. For example, Cheng and colleagues designed a new 3D complex facial expression recognition system to train children for emotional facial recognition with high-functioning ASD (aged approximately 9 to 12 years) [26]. A ViewPad showed 3D animated humanoids, and participants were required to identify certain facial expressions and then associate the corresponding emotions with appropriate situations. Participants in the intervention group demonstrated significant positive training effects when compared to the control group (*P*<.001) after 3 weeks of training.

In another study, Zink and her colleagues designed a mobile intervention to train a group of children aged 9 to 15 years with ASD for patient-doctor communication during their first dental visit [34]. The app used real-life pictures showing different treatment procedures. Positive results among the intervention group were reported (fewer sessions required to complete dental prophylaxis, *P*<.001) when compared to those receiving picture exchange communication system training in the control group.

Besides developing their own training apps, there were 2 studies in which the interventions used the built-in functions of mobile devices and achieved positive intervention effects. Gentry and his colleagues evaluated the use of PDA functions in Apple iPod Touch as a cognitive behavioral aid in supporting work among adults with ASD [29]. After being trained by occupational therapists, participants learned how to use the built-in functions of the device to obtain timely on-the-job assistance, including task reminders, task lists, maps, and videos as prompts for task completion. Those in the intervention group required significantly fewer cumulative job coaching hours than those in the control group during their first 12 months in their jobs (*P*=.01). Thiemann-Bourgue and her colleagues used iPads as SGDs to train preschoolers with ASD in communication behaviors [31]. Instead of directly evaluating the effectiveness of the intervention, that study employed trained peers for delivering the intervention as compared with the control group using untrained peers. The results demonstrated that those in the intervention group showed improved communication behaviors, manifested by more intentional communication (*P*<.001) and a more balanced proportion of responses and initiations during communication (*P*<.001).

Although the content design of mobile interventions was an important factor influencing the overall training effectiveness, mobile interventions targeting practical skills directly applied in daily lives, such as verbal communication, job coaching, and interview performance, were also effective for people with ASD. Hayes and colleagues used the video play function of mobile devices as the intervention tool to improve interview performance [28]. They developed a new app called VidCoach to train adolescents with ASD for interview skills using two training strategies, video modeling and video prompting. The intervention brought positive results, and those in the intervention group showed significantly greater improvement in interview performance than those in the control group (difference between the pretest and posttest scores: 0.561 vs 0.194, *P*<.001). In another study [30], Novack et al developed a new mobile app called Camp Discovery to improve the receptive language skills among children with ASD. This app aimed to enhance receptive language skills among younger children aged 1 to 8 years. Participants were required to correctly touch the pictures described in the voice output of an iPad. After training for a month, those in the intervention group demonstrated a significant improvement in receptive language skills than those in the delayed treatment group (*P*<.001; effect size d=2.33).

On the other hand, 4 studies reported insignificant training effects using mobile interventions targeting people with ASD. These studies used “in-app” mini games to train people for certain microskills such as visual motor, social communication, attention, and imitation skills required in social situations or performing cognitive tasks. For example, Esposito and his colleagues developed an app with interactive games to improve vocabulary, attention, and imitation skills among children with ASD [27]. Participants in the intervention group demonstrated no statistically significant improvements (*P* values ranged from.06 to.84) after the 4 weeks of training.

More importantly, a common characteristic of these 3 “insignificant” studies was the use of validated scales as the tools for effectiveness assessments. These authors administered relevant scales at baseline and postintervention and calculated the score changes to determine the intervention effectiveness. The Therapy Outcomes By You app was evaluated in 2 studies that aimed to improve language and social abilities among children with ASD [32,35]. Although the duration of the 2 interventions (3 months vs 6 months) and the level of support (support over telephone every 2 weeks vs only technical support when necessary) were varied, both studies did not achieve any significant improvements in their primary outcomes after the interventions (Whitehouse et al: *P*s=.03 to.89; Parsons et al: *P*s=.03 to.84).

In another study, Fletcher-Watsons and her colleagues developed an app called FindMe for developing social communication skills among autistic children aged under 6 years [33]. The intervention consisted of interactive games to train these children in attending to persons and following social cues. After 2 months of training with the app through an iPad, there was no significant improvement observed in the main social communication outcomes when compared with the outcomes in the control group (*P*=.29 to.74). These results suggested that after delivering the intervention to the participants through the apps, their learning might not be fully reflected in the assessment conducted using validated rating scales, when compared to measuring outcomes via actual observation or based on game performances in the apps.

## Discussion

### Principal Findings

This review identified 10 RCT studies that employed mobile technology for delivering interventions to improve the social and cognitive skills of people with ASD. Among these, 5 RCTs focused on social skills, 2 on cognitive skills, and the remaining 3 on both. Overall, most of the studies showed that mobile technology interventions could provide positive and significant training effects to improve the social and cognitive skills for people with ASD. The present review also suggests that beneficial effects are more likely to be achieved when interventions focus more on training for practical skills, such as interview skills [28], receptive language skills [30], and those providing on-the-job support [29]. The current findings provide some potential useful directions for future intervention studies.

In addition, we found that the age of the target population could potentially affect the intervention outcomes. All 4 studies recruiting adolescent or adult participants reported positive training effects, compared with the insignificant findings reported in 4 out of the 6 studies conducted with younger children aged under 6 years. This difference can be related to the fact that older participants have more “hands-on experience” and are more competent in using mobile devices, which in turn make them more receptive to mobile interventions. Therefore, mobile technology interventions are more likely to be effective among those with ASD at or beyond early adolescence.

In contrast to using mini games, training this group of people to develop more explicit and practical skills for application in real life could be easier. Similarly, the use of real pictures and 3D animation in apps might further enhance their learning. These findings suggest that the training context within the mobile intervention is crucial to the overall effectiveness of the intervention. Within a familiar context, people with ASD can perform better. In addition, practice can have a major impact on their task performance as well, and they are able to perform better in tasks with fewer modifications or unexpected changes.

It should be noted that effective interventions through mobile devices were not limited to the use of tailor-made training apps. Using the PDA [29] or speech-generating functions [31] of mobile devices, these interventions could produce significant effects among their participants, including adults and young children. One advantage of using mobile phones is the possible delivery of multisensory outputs including visual, auditory, and even vibrating stimuli, which effectively strengthen the learning process among people with ASD. In addition, some PDA functions, such as reminder alarms, are also useful solutions to improve their executive functioning and planning abilities. Therefore, these results provide further supportive evidence that mobile devices could be a favorable intervention tool for people with ASD [4-6].

Nonetheless, different assessment methods could cause substantial differences in the results. Among the studies with “ineffective” training effects [32,33,35], the assessments of the interventions (delivered through interactive games or activities in their self-designed apps) were conducted using validated scales measuring the improvements in the targeted microskills in the participants’ own lives, such as visual motor, attention, or social communication skills. Therefore, performance outcomes depended on how well participants could generalize what they had learned in the apps to their daily lives. According to the weak central coherence hypothesis [15], this skill generalization process is particularly challenging among people with ASD. Some insignificant results might not be attributed directly to ineffective training, but rather to the difficulty in applying the skills learned by the people with ASD in their daily life [33]. Given these findings, to measure the effectiveness of the intervention comprehensively, multiple assessment approaches are recommended in future studies, which should include the measures of “in-app” task performances and those used to assess how well the participants can apply these skills in real-life scenarios.

In previous systematic reviews on mobile interventions targeting people with ASD that reported significant effects [16-18], most of the included studies employed a small sample size and did not include a control group. The current findings were able to provide further positive support to the effectiveness of using mobile interventions targeting people with ASD. As a result, the robustness of these findings suggested that mobile phones could be an effective medium of intervention. In addition to other interventions shown to be effective in the past reviews, such as peer-mediated [36] or psychosocial interventions [37], mobile interventions could be another potentially viable treatment option for people with ASD.

In addition to these intervention effects, several reviewed studies further reported positive feedback from participants with ASD and their parents regarding the use of mobile devices as the means of intervention. For example, most of the participants reported higher motivation to participate in the intervention [26,27,32]. This additional benefit may not be readily achieved during interventions delivered by “real persons” in which more time and effort would be required to build a rapport and trustful relationships with participants having ASD. Therefore, even in the traditional face-to-face intervention, adding some components of mobile technology might potentially benefit the intervention effects.

According to a recent World Health Organization guideline on the use of technology to assist the delivery of health care services, “health content,” “digital health interventions,” and “digital applications” are the 3 components for effective digital health implementation [38]. The current findings state that mobile devices could serve as an effective “digital medium” for people with ASD. The guideline recommended that providing training in an internet-based setting should “complement rather than replace” [38] traditional face-to-face format, and mobile phones could be used as an additional medium of instruction, termed “mobile learning” [38]. Therefore, the overall positive findings from this review emphasize the usefulness of mobile learning for people with ASD.

### Limitations

There are several limitations that should be considered when interpreting the results of this study. First, given that mobile technology intervention is still a new research area, there were only 10 eligible RCT studies included for this review. More RCT studies with rigorous research designs would be needed to understand the actual effectiveness of these interventions. Second, as aforementioned, the previous interventions focused on different treatment outcomes and used assessment strategies. Only 2 studies used the same measurement tool; therefore, meta-analysis was not performed, which might otherwise provide a biased conclusion given the small number of studies.

### Implications for Future Studies

People with ASD have their own cognitive styles and ways of learning. The results of the present review provided several useful implications for designing future interventional studies targeting this unique population. First, mobile technology could be a promising means of providing interventions for people with ASD. Second, older participants (eg, aged above 9 years) are more likely to benefit from using mobile technology as the medium of intervention. Third, in-session training regarding the use of these apps may be the preferred mode of intervention delivery. Fourth, training materials should resemble what the participants might encounter in daily life, such as the use of real-life pictures shown on mobile devices or 3D animations created based on real faces or objects. Fifth, given the weakness of people with ASD for developing new skills, the targeted skills should be practical and related to their daily life. Finally, effectiveness assessment should be composed of different levels, such as in-app task performances and the improvement demonstrated in daily life based on self-reports or caregivers’ observations.

## Abbreviations

ASD: autism spectrum disorder
PDA: personal digital assistant
RCTs: randomized controlled trials
SGDs: speech-generating devices
